# Selective cellular imaging with lanthanide‐based upconversion nanoparticles

**DOI:** 10.1002/jbio.201800256

**Published:** 2019-01-02

**Authors:** Padmaja P. Nampi, Alexander Vakurov, Lewis E. Mackenzie, Nigel S. Scrutton, Paul A. Millner, Gin Jose, Sikha Saha

**Affiliations:** ^1^ School of Chemical and Process Engineering, Faculty of Engineering University of Leeds Leeds LS2 9JT UK; ^2^ School of Biomedical Sciences, Faculty of Biological Sciences University of Leeds Leeds LS2 9JT UK; ^3^ School of Chemistry University of Leeds Leeds LS2 9JT UK; ^4^ Department of Chemistry Durham University Durham UK; ^5^ Manchester Institute of Biotechnology and School of Chemistry University of Manchester Manchester UK; ^6^ Leeds Institute for Cardiovascular and Metabolic Medicine (LICAMM), Faculty of Medicine and Health University of Leeds Leeds LS2 9JT UK

**Keywords:** cellular imaging, luminescence upconversion, nanomaterials, toxicity, upconversion nanoparticles

## Abstract

Upconversion nanoparticles (UCNPs) with sodium yttrium fluoride, NaYF_4_ (host lattice) doped with Yb^3+^ (sensitizer) and Er^3+^ (activator) were synthesized via hydrothermal route incorporating polyethyleneimine (PEI) for their long‐term stability in water. The cationic PEI‐modified UCNPs with diameter 20 ± 4 nm showed a zeta potential value of +36.5 mV and showed an intense, visible red luminescence and low‐intensity green emission with 976 nm laser excitation. The particles proven to be nontoxic to endothelial cells, with a 3‐(4,5‐dimethylthiazol‐2yl)‐2,5‐diphenyltetrazolium bromide (MTT) assay, showing 90% to 100% cell viability, across a wide range of UCNP concentrations (0.3 ng/mL‐0.3 mg/mL) were used in multiphoton imaging. Multiphoton cellular imaging and emission spectroscopy data reported here prove that the UCNPs dispersed in cell culture media are predominantly concentrated in the cytoplasm than the cell nucleus. The energy transfer from PEI‐coated UCNPs to surrounding media for red luminescence in the biological system is also highlighted with spectroscopic measurements. Results of this study propose that UCNPs can, therefore, be used for cytoplasm selective imaging together with multiphoton dyes (eg, 4′,6‐diamidino‐2‐phenylindole (DAPI)) that are selective to cell nucleus.

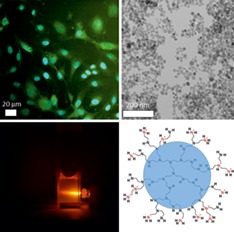

## Graphical abstract

Highly fluorescent water‐dispersible polyethyleneimine‐coated NaYF_4_/Yb^3+^/Er^3+^ upconversion nanoparticles synthesized using hydrothermal method are promising nontoxic biocompatible materials for imaging cellular cytoplasm.

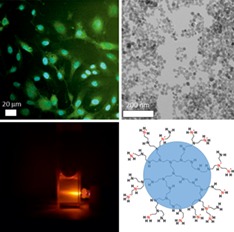

## 1 INTRODUCTION

Upconversion is a nonlinear optical process in which multiple photons in the near infrared (NIR) wavelength are absorbed by certain rare earth (RE) doped materials, which then emit photons at visible wavelengths via luminescence 1. RE‐doped upconversion nanoparticles (UCNPs) have emerged as a new class of inorganic optical probes that might overcome some of the shortcomings of fluorescent proteins and quantum dots 2. UCNPs have advantageous properties associated with their infrared excitation, including low toxicity, no visible autofluorescence or photobleaching and excellent photostability 3. The infrared excitation of UCNPs also enables imaging through up to 2 cm of tissue due to lower scattering and absorption by tissue at these wavelengths 4. Furthermore, UCNP emission can be tuned by varying elemental composition to enable multiplexed measurement 2, 5, 6, 7, 8, 9, 10, 11, 12, 13, 14, 15, 16, 17.

To meet the needs in advanced biomedical and environmental applications, several experimental methods have been developed in order to synthesize UCNPs 18, 19, 20, 21, 22, 23, 24, 25, 26, 27, 28, 29, 30, 31, 32, 33. The hydrothermal method and associated modifications provide several ways to synthesize UCNPs 5, 6, 7, 12, 13, 34, 35. Recently, imaging using luminescent UCNPs as a fluorophore alternative has been reported as a unique approach for visualizing morphological details in tissue at subcellular resolution with no visible autofluorescence and has become a powerful noninvasive tool for bioimaging 3, 5, 12, 34. Because of the inherent high photostability and nonblinking emission behavior, UCNPs have been shown to enable reliable molecular imaging with long time tracking capability 12, 35. Zijlmans et al 36 first exploited the upconversion properties of lanthanide‐doped particles for high‐performance bioimaging applications. It has been shown by Yu et al 37 that upconversion‐based visualization has negligible fading effect over time. Yu et al [37], Chatterjee et al [38], and Xiong et al [39] have established UCNPs as luminescent labels for bioimaging in living cells, and Prasad et al 40 reported in vivo imaging with Tm^3+^ as well as Yb^3+^ doped nanophosphors. High‐contrast cellular imaging has been reported using NIR to visible and NIR to NIR UCNPs 38, 41, 42. Initial attempts have been made in using UCNPs in the imaging of certain cancer cells 38, 41, 42, 43.

In the present study, water‐dispersible polyethyleneimine (PEI)‐modified NaYF_4_/Yb^3+^/Er^3+^ UCNPs have been synthesized. In addition to adopting hydrothermal method, the purification process that we followed is important to achieve highly homogeneous aqueous suspension of nanoparticles with less aggregates reported here. The purification involved ultrasonication and redispersion in deionized water followed by filtering through a PD10 column. Although addition of just PEI and use of hydrothermal method have been adopted before, in the present study, we have optimized the processing time, purification and dispersion procedures to get a stable suspension, which are very important to get a stable colloidal suspension for further applications. These PEI‐modified UCNPs, we prepared in this study, were assessed with respect to size distribution, zeta potential, crystallinity and luminescence. PEI‐modified UCNPs were used for multiphoton imaging within homogenized liver tissue and endothelial cells. The possible mechanism of luminescent resonance energy transfer (LRET) which is the reason for variation in fluorescence intensities in the visible spectrum in the biological system is discussed in the paper. We also demonstrated that the UCNPs synthesized in this study cause little tissue and cell toxicity and are mainly taken up into the cell cytoplasm. This is promising for site‐selective imaging applications of PEI‐modified UCNPs.

## 2 EXPERIMENTAL SECTION

### 2.1 Materials

Analytical grade branched PEI (Sigma Aldrich, UK, Molecular weight 25 KDa) and all other reagents including Y(NO)_3_.6H_2_O, Yb(NO)_3_.5H_2_O, Er (NO)_3_.5H_2_O, NH_4_F, NaCl, ethylene glycol (EG) and acetone were purchased from Sigma Aldrich, UK. Ultrapure (*R* = 18 MΩ) water was used for final washing of the precipitate and dispersion of the nanoparticles.

### 2.2 Nanoparticle synthesis

The experimental procedure used for UCNP synthesis was a modification of the synthesis method reported by Zhang et al 44. NaCl (10 mmol), Y(NO)_3_.6H_2_O (3.12 mmol), Yb (NO)_3_.5H_2_O (0.8 mmol), Er (NO)_3_.5H_2_O (0.08 mmol) and 1.6 g of PEI were dissolved in 60 mL EG by stirring for 2 hour to form an RE solution. About 16 mmol NH_4_F was dissolved in 40 mL of EG was prepared separately and was added to the mixed RE solution containing NaCl solution and PEI. The resultant solution was stirred for a further 15 minutes. The whole mixture was then transferred to a 120 mL Teflon‐lined Parr pressure vessel and hydrothermally heated at 200°C for 2 h. The resultant solution was then allowed to cool down to room temperature, and the contents including a very fine precipitate consisting of UCNPs were transferred into a beaker before washing 3 to 4 times with acetone, and then 4 to 5 times with ultrapure water by repeated ultracentrifugation at 80 000 times gravity for 30 minutes using a Beckman Avanti J20XP high‐speed centrifuge (Fullerton, California, USA). The nanoparticle pellet obtained was redispersed by sonication using an ultrasonic probe for a maximum of 30 seconds (Bandelin GM2070 with 100% power; cycle 0.7 seconds). The purified nanoparticles were resuspended in 4 to 5 mL of water and passed through a desalting column (PD10; GE Healthcare Life Sciences, Pharmacia Biotech Inc., UK) to separate the aggregated and finer “UCNPs.” The following sections exclusively discuss the NaYF_4_/Yb^3+^/Er^3+^ UCNPs modified with PEI thus obtained.

### 2.3 Transmission electron microscopy and dynamic light scattering (DLS)

High‐resolution transmission electron microscopy of the sample was performed using a TecnaiG2 high‐resolution field‐emission transmission electron microscope (HRFE‐TEM). The sample for the TEM was prepared by placing a drop of UCNPs suspended in water onto the surface of a holey carbon‐coated Cu grid and letting the water evaporate prior to imaging. The size distribution of nanoparticles was estimated from TEM images by using a custom MATLAB algorithm (MATLAB 2016a), based upon a circular Hough transforms to detect the pseudo‐spherical UCNPs 45. The diameter of the nanoparticle in pixels was subsequently converted to nanometer (nm) by known scale calibration of the HRFE‐TEM. Selected area electron diffraction (SAED) was used to verify the lattice planes by comparing with known reference patterns. Energy‐dispersive X‐ray (EDX) analysis of the sample was also performed during HRFE‐TEM to confirm the elemental composition.

DLS was used to ascertain the hydrodynamic diameter and zeta potential of the UCNPs when dispersed in water. Malvern Zetasizer Nano Zs system (Malvern, UK) was used for the DLS measurement.

### 2.4 X‐ray diffraction measurement

X‐ray diffraction (XRD) measurements of the sample powder obtained by drying the sample at 100°C were made with an X‐ray diffractometer (Bruker D4) using Cu Kα radiation (*λ* = 1.5418 A**°**) in the 2θ range 10**°** to 80**°** in 0.0247 increments.

### 2.5 Luminescence emission spectroscopy

In order to measure the luminescence, the sample solution was loaded into 2.0 mL quartz Suprasil cuvettes (Hellma Analytics, UK) placed within a cuvette holder (qpod 2e, Ocean Optics Inc., Liberty Lake, Washington, USA) at room temperature. The sample was illuminated with a 976 nm NIR laser (BL976‐PAG900; Thorlabs, New Jersey, USA), operating at a power corresponding to 1000 mA current. UCNP emission was recorded using a high‐performance spectrometer (QE‐PRO, Ocean Optics, Florida, USA) with 1‐second integration time and no data averaging.

### 2.6 In vitro endothelial cell culture

Human umbilical vein endothelial cells (HUVECs; PromoCell, Germany) were used for the in vitro cell study. Passage of cells was carried out by standard trypsinization (Sigma Aldrich, UK). After centrifugation, the pellet was resuspended and cells counted using a hemocytometer. HUVECs (1 × 10^4^) were grown overnight in a 96‐well plate. Growth media were prepared using a PromoCell endothelial cell growth medium (PromoCell GmbH, Heidelberg, Germany) supplemented with PromoCell endothelial cell supplements (PromoCell GmbH, Heidelberg) and 10% (v/v) Gibco fetal calf serum and used to feed the growing cells every 2 days until the cells reached ~90% confluency. Media were filtered using Millipore Express polyethersulfone (PES) membranes (pore size 0.22 μm, diameter 33 mm, sterile; γ‐irradiated, UK) and Terumo syringes (Terumo, UK).

### 2.7 In vitro cytotoxicity evaluation

The in vitro cell viability of UCNPs within endothelial cells was assessed using the 3‐(4,5‐dimethylthiazol‐2yl)‐2,5‐diphenyltetrazolium bromide (MTT) (Sigma Aldrich, UK) assay. Briefly, 10^4^ HUVECs were grown overnight in a 96‐well plate. Growth medium was prepared using a PromoCell endothelial cell growth medium supplemented with PromoCell endothelial cell supplements and 10% (v/v) fetal calf serum. Increasing doses of nanoparticles from 0.3 ng/mL to 300 μg/mL in growth media were added and incubated for a period of 24 hours. The MTT assay was carried out after 24 hours by adding 50 μL of 5 μg/mL MTT to each well. After the addition of MTT, the plate was incubated for a period of 3 hours, until the purple product appeared. Then, the purple crystals were dissolved using 300 μL/well of isopropanol, and the plate was read at 576 nm. Cell morphology was observed under light microscope (Olympus BX41, Japan) using Cell F imaging software for Life science microscopy in order to examine any change after addition of nanoparticles.

### 2.8 In vivo toxicity testing for liver tissue

All experiments were performed on 10‐week‐old, 25 g to 30 g male C57BL/6J mice (Harlan Olac, Bicester, UK), under appropriate United Kingdom Home Office personal and project licenses, adhering to the regulations as specified in the Animals (Scientific Procedures) Act (1986), and according to institutional ethical guidelines. The mice were anesthetized with isoflurane (1%‐1.5% in oxygen). The test mice were injected with 100 μL of 0.2 mg/mL of UCNPs in 0.1 M phosphate buffer saline (PBS, pH 7.6) intraperitoneally. Control mice were injected with 100 μL of PBS (pH 7.6). Following 48 hours, both experimental and control mice were perfused transcardially with PBS followed by 4% paraformaldehyde in 0.1 M phosphate buffer (pH 7.4). Liver tissue was harvested and stored in PBS until further processing. For in vivo toxicity assessment, a part of the liver was fixed in paraformaldehyde and embedded in paraffin using a MEDITE Paraffin Embedding System TES 99, GmbH, Germany. The embedded tissues were cut into thin sections using a Leica RM2235 (Leica Biosystems, GmbH, Germany) rotary microtomes and consequently processed and stained with standard hematoxylin and eosin staining. The histological sections were observed under an Olympus BX41 bright field microscope, and the digital images were monitored using Image Pro Plus 7 software (Media Cybernetics, UK) for tissue morphology to establish the nontoxicity of the nanoparticles toward tissues.

### 2.9 Multiphoton imaging of ex vivo liver tissue

Mice were killed by decapitation under anesthesia (isoflurane, 1%‐1.5% in oxygen), liver removed and kept in PBS. Part of the liver tissue was cut and homogenized with a blunt needle. About 100 μL of the UCNPs (diluted 1 μL in 10 000 μL PBS) were added to the tissue and mixed the particles with the tissue by further homogenization and vortexing. The homogenized tissue with embedded UCNPs was mounted on a slide and air‐dried in a refrigerator. The samples were coverslipped using the mounting medium containing 4′,6‐diamidino‐2‐phenylindole (DAPI) (Vectashield antifade mounting medium with DAPI, Vector Laboratories, UK) and viewed under multiphoton microscope.

### 2.10 Multiphoton imaging of endothelial cells

HUVECs were grown on small circular coverslips on an 8‐well plate in the exactly the same way as mentioned in the in vitro toxicity evaluation measurements. After the cells became confluent, 50 μL of 0.3 ng/mL of nanoparticle solution was added and incubated for 24 hours. A coverslip with cells without nanoparticle was used as the control. After incubation, the cells were fixed using 70% methanol. The coverslip with samples were mounted on a glass slide with the mounting medium containing DAPI (Vectashield antifade mounting medium with DAPI; Vector Laboratories, UK) and observed under the multiphoton microscope.

Images were acquired using a multiphoton microscope (Upright Zeiss 710; Chameleon, Coherent, Glasgow, UK) with tunable laser excitation (690‐1064 nm); 980 nm was selected as it corresponds to peak excitation of NaYF_4_/Yb^3+^/Er^3+^ UCNPs. An external nondescanned detector was used to acquire Multiphoton images (Carl Zeiss, Jena, Germany). Emission spectroscopy analysis of cells was enabled by a spectral detector with photonmultiplier tube (Carl Zeiss, Germany). All measurements were acquired at 100% excitation power, corresponding to 180 mW.

## 3 RESULTS AND DISCUSSIONS

### 3.1 Transmission electron microscopy of UCNPs

**Figure 1:**
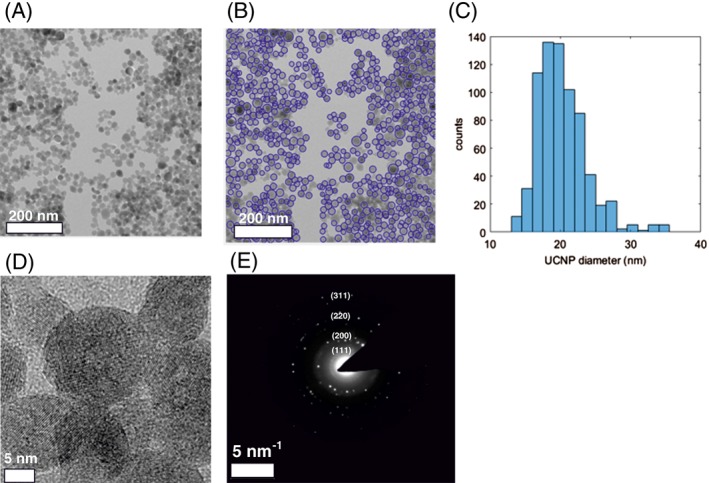
(A) TEM image of the NaYF_4_/Yb^3+^/Er^3+^ UCNPs as synthesized. (B) Detection of UCNPs for size analysis; the blue circles show nanoparticles selected for analysis. (C) Resultant size distribution, showing a normal distribution with an average UCNP diameter of 20 ± 4 nm (mean ± SD). (D) HRFE‐TEM image of UCNPs. (E) Selected area diffraction pattern demonstrating peaks from UCNP crystal planes

HRFE‐TEM images of the UCNPs showed a homogeneous distribution with an approximate spherical morphology, with an average particle diameter of 20 ± 4 nm (mean ± SD) (Figure 1A‐C). The 2D crystal lattice fringes are visible in Figure 1D; and the distance between the crystal planes are determined to be 3 and 2.6 Å, corresponding to the (111) plane and (200) plane, respectively 46. The SAED pattern (Figure 1E) demonstrates the highly ordered crystal structure of the UCNPs with the (111), (200) and (220) crystal planes clearly present. From the distance between the crystal planes, it can be inferred that UCNPs prepared in this research have α‐NaYF_4_ cubic crystal phase 20. The EDX analysis spectrum reported in Supporting Information Figure S1 indicates the molar ratio of the elements Na/Y/Yb/Er/F as 1/0.778/0.210/0.027/4.04, respectively. The concentration of nitrogen at 0.87 atom percentage indicates the presence of PEI on the surface of the particles.

### 3.2 XRD analysis of UCNPs

**Figure 2:**
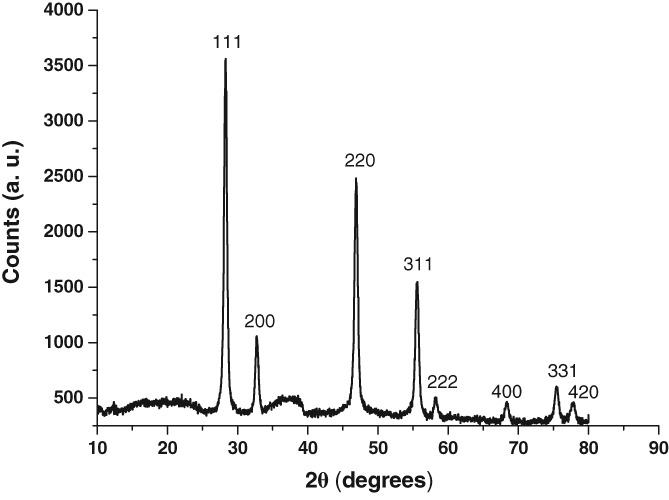
XRD pattern of UCNPs. Comparison of the distinct diffraction peaks with known reference shows that the UCNPs are of cubic crystal structure

The XRD measurements (Figure 2) also confirmed that the crystals are cubic (α‐phase) by comparison with the standard test card (JCPDS Card No.77‐2042) 20. The hkl matches with TEM data for the first three planes. From the 2θ peak positions, the cubic lattice parameter, *a* was calculated to be 5.47 ± 0.02 Å (mean ± SD calculated from six individual peaks).

### 3.3 DLS measurements (particle size in suspension and zeta potential)

**Figure 3:**
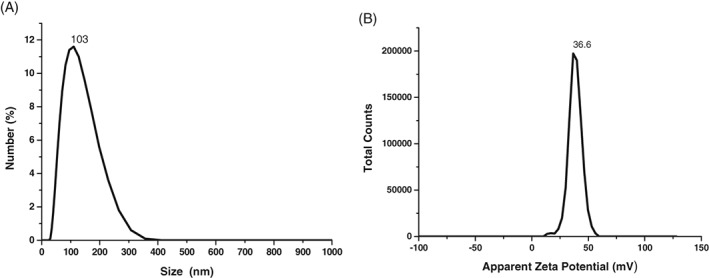
(A) DLS particle size distribution of UCNPs showing maximum particles with mean size corresponding to a hydrophobic diameter of around 100 nm. (B) Zeta potential measurement of the UCNPs showing high surface charge

Figure 3A and B shows the distribution of particles sizes of the UCNPs in solution and the zeta potential measurement respectively, measured using DLS. The particle size distribution of the UCNPs by DLS indicated that the maximum particles with mean particle diameter of around 100 nm (Figure 3A). Owing to the outer layer of PEI coating on the UCNPs, an increase in hydrodynamic diameter is expected compared with the UCNP size calculated by TEM 44. Consistent with surface modification by PEI, the PEI‐UCNP constructs showed excellent long‐term stability in water without any noticeable agglomeration. The long‐term water stability makes these PEI‐UCNPs suitable for bioimaging applications. The maximum apparent zeta potential value of +36.5 mV was found to be highly consistent with the isolation of PEI‐coated UCNPs. The value suggests that PEI‐UCNPs have cationic surfaces; this can be attributed to the −NH_2_ group of the PEI being attached to the particle surface as a hydrophilic head 44. The positive zeta potential is advantageous for two reasons: (a) improved stability in water and (b) better cell membrane permeability than negatively charged particles 47. Thus high zeta potential value is a highly favorable property for tissue and cellular imaging.

### 3.4 Luminescence emission of UCNPs

**Figure 4:**
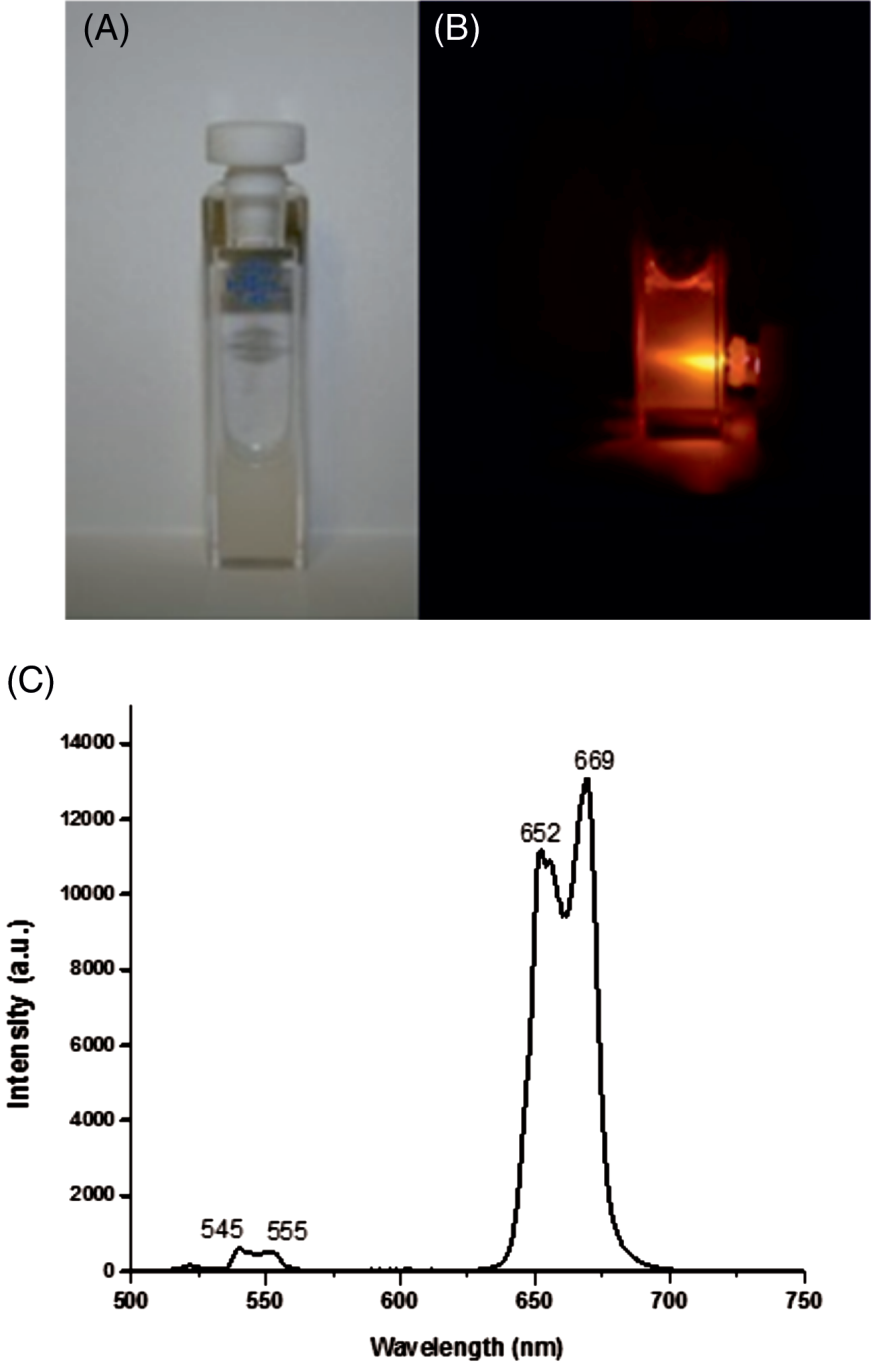
(A) Photograph of the stable UCNP dispersion without excitation in a glass cuvette. (B) UCNP luminescence under 976 nm laser excitation showing orange/red luminescence to the eyes. (C) Luminescence emission spectrum of UCNPs recorded by a spectrometer showing peaks in the green (~540 nm) and red (~660 nm) regions, with the red peak showing considerably greater intensity

Figure 4A shows the homogeneous suspension when not irradiated with 976 nm laser; the white opaque appearance is due to nonabsorptive light scattering by the PEI‐UCNPs. Under 976 nm illumination, UCNPs show apparent orange/red emission visible to the eye (Figure 4B). The UCNP luminescence emission spectrum of the suspension with 976 nm excitation is shown in Figure 4C. The sharp doublet peak in the red wavelength range (635‐694 nm) is due to the ^4^F_9/2_ → ^4^I_15/2_ transitions, while the weak emission band at 540 to 560 nm results from the (^4^S_3/2_, ^2^H_11/2_) → ^4^I_15/2_ transitions. The combination of both red and green under 976 nm NIR irradiation results in the deep orange/red emission in the visible light region as shown in Figure 4B.

### 3.5 In vitro cytotoxicity assessment in endothelial cell and in vivo liver toxicity assessment

**Figure 5:**
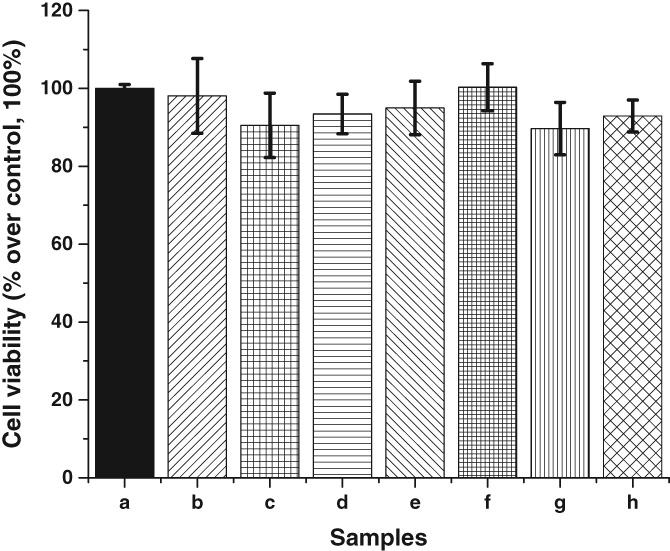
MTT cell viability assay of human endothelial demonstrating the UCNPs are not cytotoxic to endothelial cells. (a) Control (b‐h) various concentrations of UCNPs introduced to the sample; 0.3 ng/mL, 3 ng/mL, 30 ng/mL, 300 ng/mL, 3 μg/mL, 30 μg/mL and 300 μg/mL, respectively

Cytotoxicity is a concern whenever nanoparticles are applied to the imaging of cells or tissues. An MTT assay was used to assess the cytotoxicity of the UCNPs on human endothelial cells. Incubating the cells with UCNPs for 24 hours resulted in average cell viability of 90% to 100% (Figure 5), indicating that UCNPs are nontoxic to endothelial cells. Furthermore, no change in cell number or morphology was apparent with the maximum concentration of UCNPs (300 μg/mL), indicating good cell viability (Supporting Information Figure S2).

**Figure 6:**
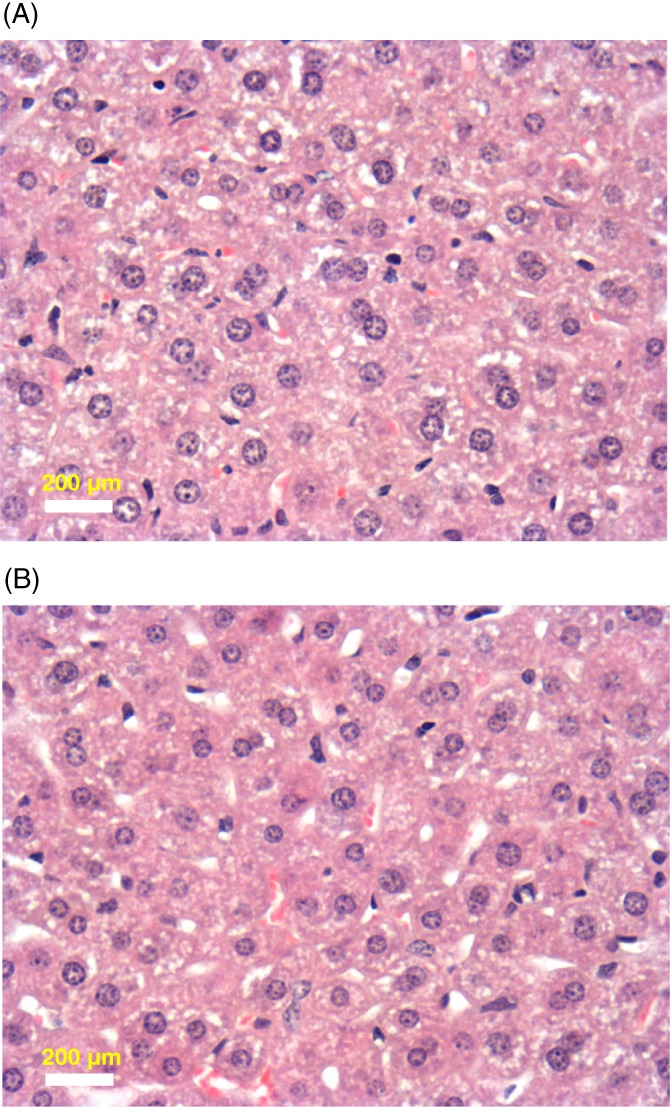
Hematoxylin and eosin stained liver tissue with and without injection of UCNPs. No apparent morphological difference between the control sample (A) and the tissue sample with UCNPs (48 hours postinjection) (B) is evident from the images

Recent work by Zou et al 48 indicates that when UCNPs are introduced to mice, the UCNPs accumulated in the liver after 24 hours. In this research, in vivo toxicity assessment of the UCNPs was carried out in mice. The microscopy images of liver tissue from mice injected with UCNPs and stained with hematoxylin and eosin shown in Figure 6 suggest no apparent tissue damage or lesions in the UCNPs incorporated tissues compared with the control. There was no visible difference in morphology between the control (Figure 6A) and the tissues with the UCNPs (Figure 6B), indicating no apparent toxicity effect of the UCNPs on liver tissues after 48 hours incubation.

### 3.6 Multiphoton microscopy of UCNPs within homogenized liver tissue and endothelial cells

**Figure 7:**
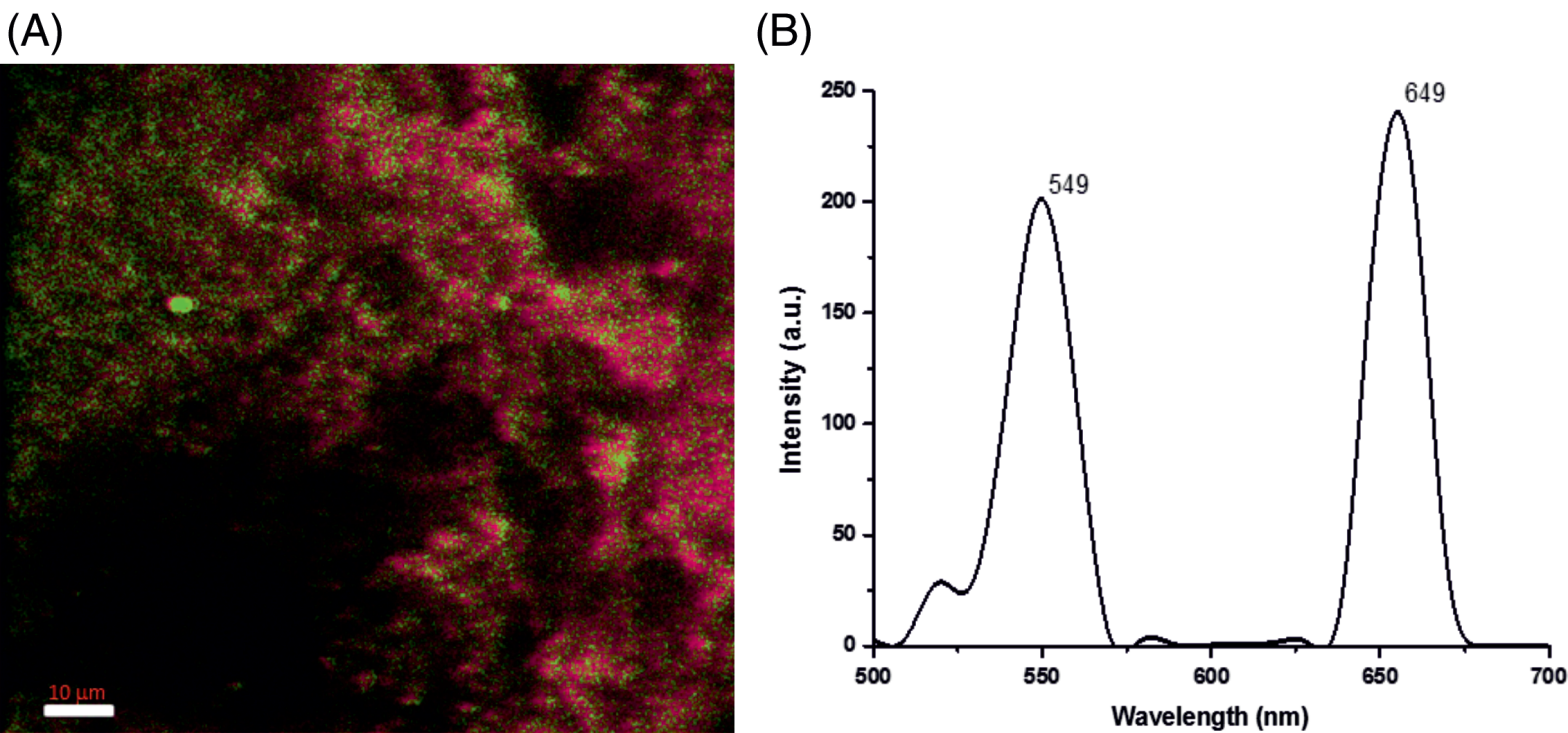
(A) Multiphoton image of the homogenized mouse liver tissue with UCNPs after 48‐hour incubation, excited at 980 nm. The reason for spatial distribution of red and green emission is currently unclear. (B) Representative λ‐scan emission spectrum of the corresponding image at 980 nm excitation clearly showing the characteristic green and red luminescence peaks of UCNPs

Figure 7A shows the multiphoton image of the liver tissue homogenized with the UCNPs, excited at 980 nm. The characteristic green and red emission luminescence spectra of UCNPs were observed in the homogenized liver tissue (see Figure 7B). The spectra are distinct from the typical liver autofluorescence, which under normal ultraviolet or visible excitation is typically a continuous peak ranging from 400 to 650 nm 49. Note, the sharp peaks of UCNPs are not clearly resolved in Figure 7B due to the low spectral sampling resolution of the multiphoton microscope (approximately 10 nm).

The spectral emission data of UCNPs in tissue shows a pseudo‐uniform distribution of particles in the homogenized tissue. The red emission peak was greatly reduced compared with the UCNPs in as prepared solution (see Figure 4C) suggesting that red emission quenching in Figure 7B is due to a localized interaction between the UCNPs and molecular constituents of liver tissues. The peak intensity ratio of red to green is 24.3 for UCNPs in solution while that in liver tissues is 1.03. The UCNP emission ratio between red and green is also nonuniform across the homogenized tissue sample though there is a drop in red intensity in comparison with green everywhere. The reason for this is due to localized interactions between the UCNPs and the variations in the molecular sites in the homogenized tissue that is attached to the UCNPs, resulting in energy loss from the UCNPs via LRET identical to that observed in RE complexes 50. These data support that UCNPs are suitable for tissue imaging without autofluorescence.

**Figure 8:**
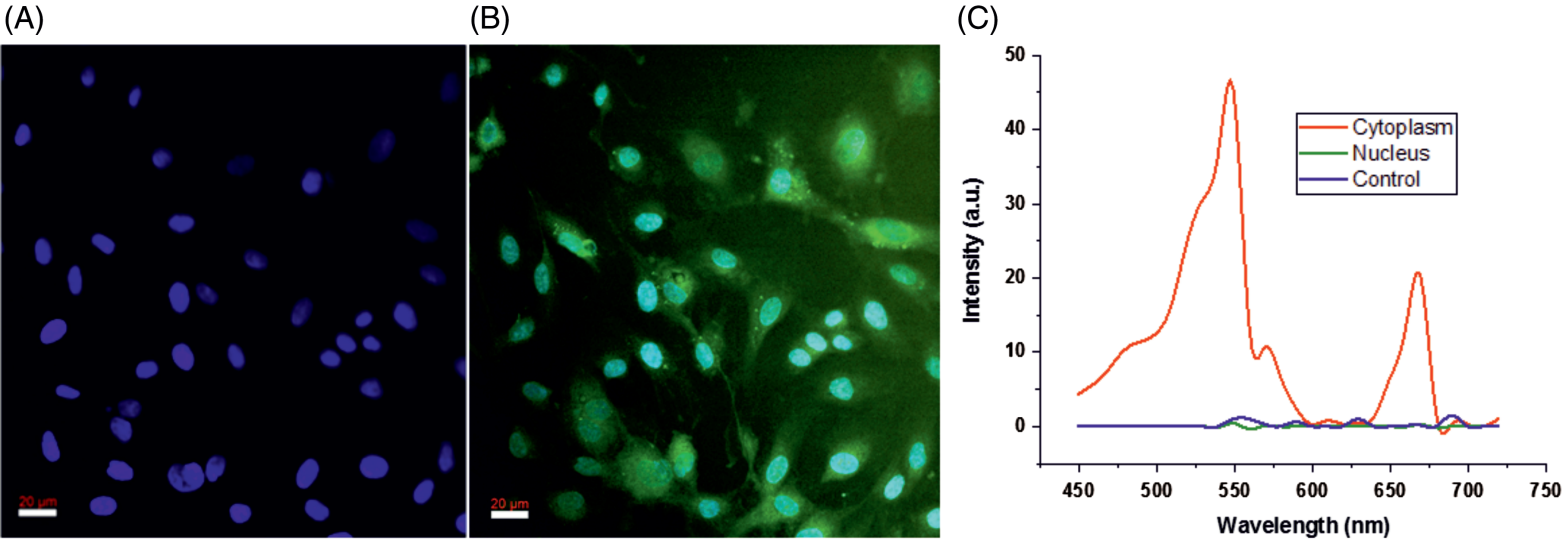
Multiphoton images of DAPI‐stained endothelial cells with low concentration of nanoparticles (0.3 ng/mL) added and subsequently incubated for 24 hours. (A) 760 nm excitation, highlighting the DAPI‐stained cell nucleus only. (B) 980 nm excitation image of excited UCNPs overlaid upon (A) showing apparent UCNP emission from cytoplasm. (C) λ‐curve showing the wavelength of emission from the cytoplasm, nucleus and control cells at 980 nm

Studies have also been conducted to see the interaction and uptake of UCNPs by endothelial cells. Figure 8A shows the multiphoton image of the endothelial cells with mounting medium containing DAPI and UCNPs when excited at 760 nm. The image clearly shows only the DAPI‐stained cell nucleus at this excitation wavelength. Figure 8B showed the overlay images obtained at 760 and 980 nm, respectively. It can be clearly seen that the particles are predominantly attached to the cells around the nucleus, and the cell morphology is not affected. This is consistent with the previous observation 51.

In order to confirm the origin of the intense green emission, the spectra were recorded from various locations within the cell media and reported in Figure 8C. The spectrum recorded in the cytoplasm clearly shows characteristic dual‐emission peak of UCNPs, whereas the nucleus shows no emission, indicating UCNPs are only absorbed into the cytoplasm and not into the nucleus. Figure 8B,C shows that UCNPs are localized in the cytoplasm but not in the nucleus. This clearly demonstrates the importance of UCNPs in the selective multiphoton imaging of cellular cytoplasm. The wavelengths of emission from the cytoplasm (Figure 8C) also show relatively higher emission intensity in the green region than the red region. The ratio of red to green (R/G) peak intensity in this spectrum is 0.18, which is significantly lower than that of UCNPs in solvent reported in Figure 4C. The quenching of red emission could be attributed to the LRET from the UCNPs to the cellular proteins, located within 20 nm of UCNPs similar to that described above for UCNPs in liver tissues. However, in both cases, a detailed understanding of the LRET requires knowledge of the molecules in the biological media that preferentially attaches to UCNPs and time‐resolved spectroscopic studies. Figure 8 also demonstrates that DAPI can be used in conjunction with UCNPs for dual wavelength excitation for selective imaging of nucleus and cytoplasm, respectively. Results reported here are promising for using the PEI‐coated UCNPs safely in multiphoton bioimaging applications with multiple wavelength imaging modality.

## 4 CONCLUSIONS

PEI‐modified NaYF_4_/Yb^3+^/Er^3+^ UCNPs were synthesized by a novel hydrothermal method. HRFE‐TEM showed particles with a mean diameter of 20 ± 4 nm and an overall diameter range between 10 and 35 nm. Measurements with DLS indicated a much wider diameter distribution when in solution, with a mean nanoparticle diameter of ~100 nm due to the hydrated PEI outer layer. The UCNP zeta potential value of +36.5 mV indicates the cationic surface of the particles and PEI modification increased the water stability of the nanoparticles; an important parameter for cellular uptake in bioimaging applications. The nontoxicity of the particles toward liver tissue and endothelial cells was confirmed by hematoxylin and eosin staining and MTT assays, respectively. Imaging of UCNPs in the presence of homogenized liver tissue exhibited the characteristic luminescence spectra of UCNPs, indicating that UCNPs can be used for tissue imaging without background tissue autofluorescence. Multiphoton imaging and emission spectroscopy of endothelial cell utilizing UCNPs indicated that UCNPs are absorbed into the cytoplasm, but not to the nucleus. This indicates that UCNPs are suitable for cell structure applications and that our PEI‐modified UCNPs were uptaken into the cytoplasm specifically. This initial study demonstrates that in the future, UCNPs could be applied to bioimaging of cell structures and tissue imaging. LRET, which is the main cause of drop in luminescence intensity of red relative to green for UCNPs in biological media, is observed in this research. With appropriate surface modification and suitable bioconjugation procedures, UCNPs could be utilized for targeted imaging and as biosensors for sensing of biomolecules and proteins.

## AUTHOR BIOGRAPHIES

Please see Supporting Information online.

## Supporting information


**Author Biographies**

**Figure S1.** EDX of the UCNP prepared which establishes the molar ratio of the elements Na/Y/Yb/Er/F as 1/0.778/0.210/0.027/4.04.
**Figure S2.** Optical transmission microscopy of endothelial cells (A) Before UCNP addition (B) After addition of 300 μg/mL UCNPs and incubation for 24 hours.
