# Supplementary material for: Selective cellular imaging with lanthanide‐based upconversion nanoparticles
Source: J Biophotonics. 2019 Jan 2;12(4):e201800256. doi: 10.1002/jbio.201800256 (PMC7065621; doi:10.1002/jbio.201800256)
Supplement: Supplementary file 1 — Author Biographies [file JBIO-12-e201800256-s002.docx]

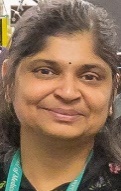


Dr. Padmaja P. Nampi received her PhD. in 2003 from National Institute for Interdisciplinary Science and Technology of the Council for Scientiﬁc and Industrial Research (CSIR), India (Mahatma Gandhi University, Kerala, India). She worked as Postdoctoral Research Scientist at the National Institute of Advanced Industrial Science and Technology (AIST), Nagoya, Japan from 2004 to 2006. She also worked as Senior Research Associate of CSIR and Principal Investigator, Department of Science and Technology, Government of India at Sree Chitra Tirunal Institute for Medical Sciences and Technology (SCTIMST) in collaboration with University of Leeds, UK. Currently, she is a Marie Skłodowska-Curie Experienced Researcher at the School of Chemical and Process Engineering, University of Leeds, UK. Her current research interests include nanomaterials, mainly upconversion nanomaterials for various biomedical applications such as biosensing and bioimaging.

Dr. Alexander Vakurov graduated in Chemistry and received his PhD. in Chemistry from Moscow State University. Currently he is a Research Fellow in the School of Chemistry at University of Leeds. His interest stays in biosensors and nanoparticles research. Presently, he is working on the development of electrochemical toxicity sensor based on the model phospholipids monolayer on mercury film electrode and fluorometric sensor based on unilamellar liposomes.

*
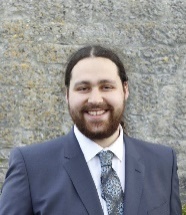
*

Dr. Lewis E. Mackenzie received his B.Sc. (Hons.) in Physics and Astronomy, and M.Sc. (Astrophysics) from the University of Glasgow in 2011 and 2012 respectively. He then perused a Ph.D. in developing novel multispectral imaging techniques for measurement of blood oxygen levels *in vivo*, graduating from the University of Glasgow in 2016. From 2016 to 2017 he was a Postdoctoral Research Fellow at the University of Leeds where he worked to develop luminescent lanthanide-based upconversion nanoparticle biosensors along with related optical readout instrumentation. Currently, he is a Postdoctoral Research Associate associated at Durham University, where he develops instrumentation to measure circularly polarised luminescence from chiral lanthanide complexes.

**
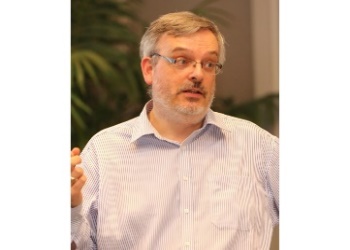
**

Prof. Nigel S. Scrutton is Professor and Director of the Manchester Institute of Biotechnology, University of Manchester, UK. He is also Director of the Manchester Centre for Synthetic Biology (SYNBIOCHEM). He obtained his Ph.D. and Sc.D from the University of Cambridge, UK. He has held a number of academic and fellowship positions prior to taking his current position at the University of Manchester. He has interests in enzyme biophysics and structure, and enzyme catalysis and design.


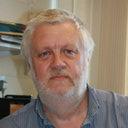


Prof. Paul A. Millner is a Professor in Bionanotechnology at the School of Biomedical Sciences of Faculty of Biological Sciences at University of Leeds, UK. He obtained his PhD. from University of Leeds and worked in Purdue University, USA and Imperial College, London, before joining as a Faculty in Leeds. He is an expert in applying nanobiotechnolgy concepts for various advanced applications. His main interests and expertise include targeted immobilization of proteins and the use of affinity reagents to direct self-assembly of biosensor surfaces, and construction of organic, polymeric, and inorganic nanoparticulate biocatalysts and biosensors.

**
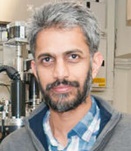
**

Prof. Gin Jose received his MSc. and PhD. degrees in Physics from University of Calicut, India and Mahatma Gandhi University, Kottayam. India in 1996 and 2003 respectively. He was a Research Fellow at the Dipartimento do Fisica, Politecnico di Milano, Milan, Italy during 2001-2002. He was Senior Lecturer/Assistant Professor in Physics at the Indian Institute of Technology, Guwahati during 2003-2007. In   2007,   he   joined   the School of Chemical and Process Engineering at University of Leeds, UK and since March 2013 he is the Professor and Chair in Functional Materials there. He is currently leading the research group, Applied Photon Science. Professor Jose has research experience in the area of novel laser glasses, photonic integration with dissimilar materials, glass thin films, pulsed laser processing of materials, sol-gel processing, laser spectroscopy and ion-exchange with emphasis on applications in biochemical sensors/imaging, photonic components engineering and energy applications.


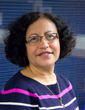


Dr. Sikha Saha is an Associate Professor of Cardiovascular Medicine at the University of Leeds. Her research interests include biosensor development, drug delivery, metabolic profiling and toxicity testing in cardiovascular and neurological diseases. She has extensive experience in *quasi vivo* blood brain barrier model, lanthanides and nanoparticles based biosensing and bioimaging, and preclinical testing of novel drugs and diagnostic devices using *in vitro* and *in vivo* mammalian models.
