# Supplementary material for: Selective cellular imaging with lanthanide‐based upconversion nanoparticles
Source: J Biophotonics. 2019 Jan 2;12(4):e201800256. doi: 10.1002/jbio.201800256 (PMC7065621; doi:10.1002/jbio.201800256)
Supplement: Supplementary file 2 — Figure S1. EDX of the UCNP prepared which establishes the molar ratio of the elements Na/Y/Yb/Er/F as 1/0.778/0.210/0.027/4.04. Figure S2. Optical transmission microscopy of endothelial cells (A) Before UCNP addition (B) After addition of 300 μg/mL UCNPs and incubation for 24 hours. [file JBIO-12-e201800256-s001.docx]

Supporting Information

**Selective cellular imaging with lanthanide-based upconversion nanoparticles**

*Padmaja P. Nampi^1,^*, Alexander Vakurov^2,3^, Lewis E. Mackenzie^2,4^, Nigel S. Scrutton^5^****,*** *Paul A. Millner^2^_,_ Gin Jose^1^, Sikha Saha****^6^***

^1^School of Chemical and Process Engineering, Faculty of Engineering, University of Leeds, United Kingdom, LS2 9JT

^2^School of Biomedical Sciences, Faculty of Biological Sciences, University of Leeds, United Kingdom, LS2 9JT ; ^3^Present address: School of Chemistry, University of Leeds, United Kingdom, LS2 9JT; ^4^Present address: Department of Chemistry, Durham University, Durham, United Kingdom, DH1 3LE

^5^Manchester Institute of Biotechnology and School of Chemistry, University of Manchester, United Kingdom, M1 7DN

^6^Leeds Institute for Cardiovascular and Metabolic Medicine (LICAMM), Faculty of Medicine and Health, University of Leeds, United Kingdom, LS2 9JT

* Corresponding Author: E-mail: [p.nampi@leeds.ac.uk](mailto:p.nampi@leeds.ac.uk)


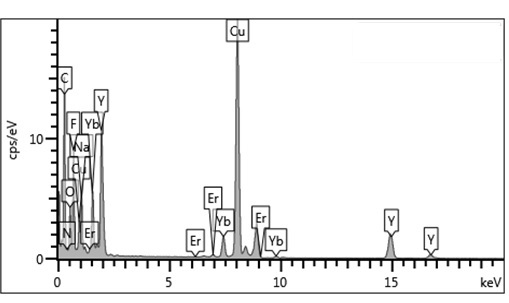


**Figure S1:** EDX of the UCNP prepared which establishes the molar ratio of the elements Na/Y/Yb/Er/F as 1/0.778/0.210/0.027/4.04


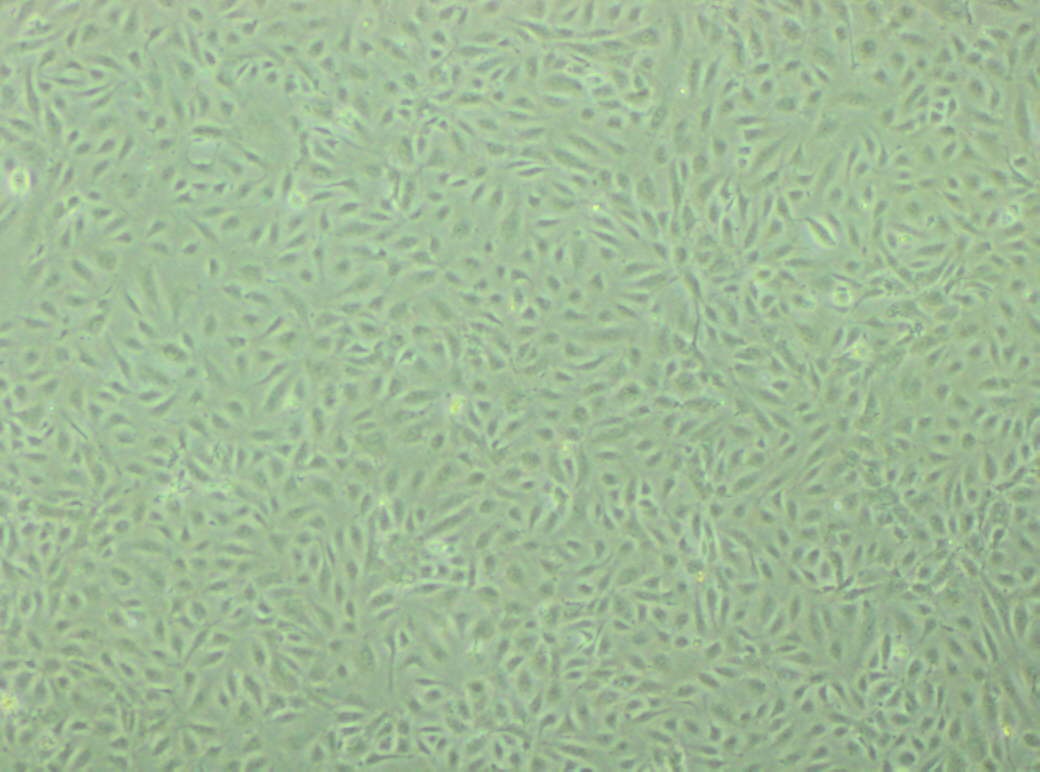

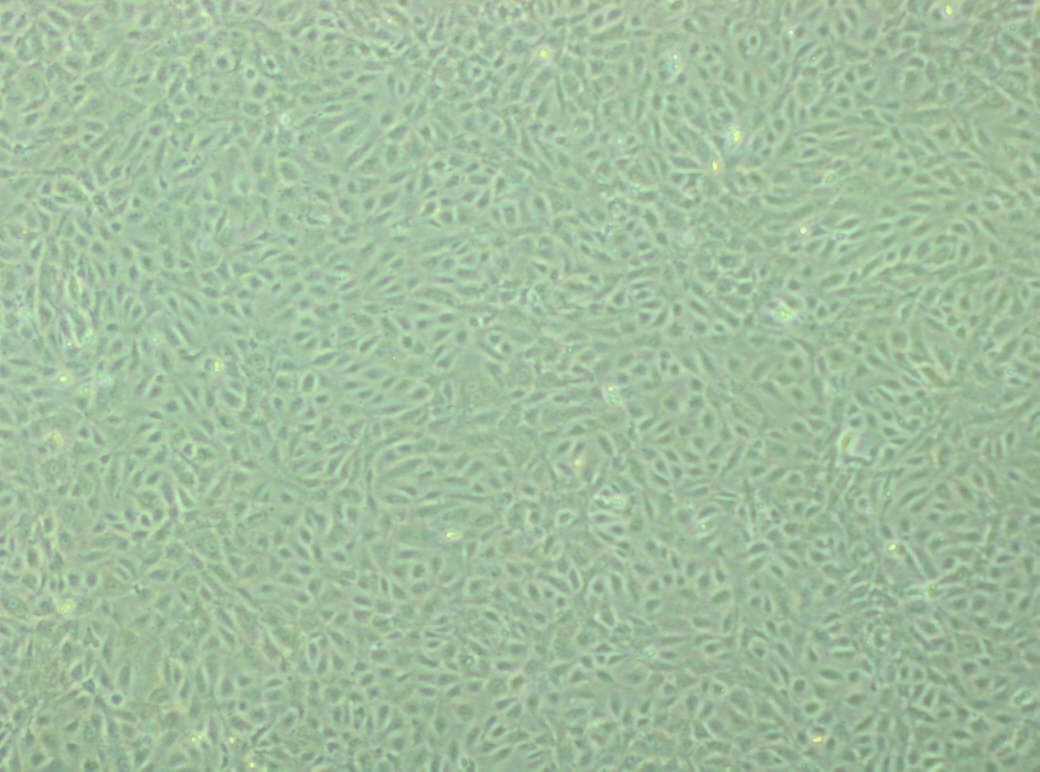


100µm

100µm

A

B

**Figure S2:** Optical transmission microscopy of endothelial cells (A) Before UCNP addition (B) After addition of 300 μg/mL UCNPs and incubation for 24 hours.
